# Activation of GABA(A) receptors inhibits T cell proliferation

**DOI:** 10.1371/journal.pone.0251632

**Published:** 2021-05-20

**Authors:** Emma L. Sparrow, Sonya James, Khiyam Hussain, Stephen A. Beers, Mark S. Cragg, Yury D. Bogdanov

**Affiliations:** Antibody and Vaccine Group, Centre for Cancer Immunology, MP127, University of Southampton Faculty of Medicine, Southampton, Hants, United Kingdom; Indiana University School of Medicine, UNITED STATES

## Abstract

**Background:**

The major sites for fast synaptic inhibition in the central nervous system (CNS) are ion channels activated by γ-aminobutyric acid (GABA). These receptors are referred as GABA(A) receptors (GABA(A)R). Recent evidence indicates a role of GABA(A)R in modulating the immune response. This work aimed to discern the role of GABA and GABA(A)Rs in human and mouse T cell activity.

**Methods:**

Mouse splenocytes or human peripheral blood mononuclear cells (PBMCs) were activated with anti-CD3 antibodies and the proliferation of both CD8^+^ and CD4^+^ T cells assessed through flow cytometry. Subsequently, the effects on T cell proliferation of either GABA(A)R modulation by diazepam that is also capable of activating mitochondrial based translocator protein (TSPO), alprazolam and allopregnanolone or inhibition by bicucculine methiodide (BMI) and (1,2,5,6-Tetrahydropyridin-4-yl)methylphosphinic acid (TPMPA) were assessed.

**Results:**

Positive modulation of GABA(A)Rs either by benzodiazepines or the neurosteroid allopregnanolone inhibits both mouse and human T cell proliferation. GABAergic inhibition of T cell proliferation by benzodiazepines could be rescued by GABA(A)R blocking. Our data suggest that benzodiazepines influence T cell proliferation through both TSPO and GABA(A)Rs activation.

**Conclusions:**

We conclude that activation of GABA(A)Rs provides immunosuppression by inhibiting T cell proliferation.

## Background

The main inhibitory neurotransmitter within the CNS is GABA, acting either through ionotropic GABA(A)R or metabotropic GABA(B) receptors (GABA(B)R). GABA(A)Rs are a diverse group of proteins consisting of 19 different subunits (α1–6, β1–3, γ1–3, δ, ε, π, θ, ρ1–3). To make functional GABA(A)Rs these subunits form either pentamers consisting of two α, two β and one γ (or δ, ε, π, θ) subunits or pentamers of ρ subunits [1]. Activation of GABA(A)Rs on neurons leads to hyperpolarization of cell membranes and inhibition of action potentials [2]. GABA(A)Rs are important targets for pharmacological agents used in routine clinical practice including benzodiazepines, barbiturates, neurosteroids and certain anaesthetics [2]. They have also been implicated in various pathological conditions including anxiety, depression, epilepsy and substance abuse [3].

Although previously considered solely a regulator of neuronal behaviour, recent studies suggest a wider role of GABA outside of the CNS. It has been shown that GABA potentiates pancreatic α to β cell conversion as well as β cell proliferation [4–6], although a subsequent study did not replicate these findings [7]. Nevertheless, a clinical trial assessing the safety and effects of GABA on children with newly diagnosed Type 1 diabetes has been recently started [8]. This is based on data that GABA not only promotes pancreatic α to β cell conversion and β cell proliferation but also supresses the immune response against β cells by increasing the number of regulatory T cells (Tregs) in the spleens of GABA treated mice [9].

Various reports have also indicated the presence of components of GABAergic signalling in cells of the immune system. GABA(A)Rs were identified on T cells, B cells, macrophages and dendritic cells from humans and rodents in addition to various cell lines mostly by RT-PCR [10–13]. These reports differ in details and, therefore, require further confirmation preferably with the variety of methods. GABA producing enzymes GAD65 and GAD67 have also been found to be expressed in immune cells. GAD65 has been observed in dendritic cells and to a lesser extent in macrophages. Its expression seems to be variable and increased upon stimulation [14]. Dionisio et al. [12] identified the presence of GAD67 in various subtypes of human lymphocytes. Several effects of GABAergic stimulation on the function and characteristics of immune cells have also been reported. Modulation of GABA(A)Rs has been observed to elicit suppression of T cell proliferation [15, 16], influence mouse macrophage phenotypic polarization [17], modulate the production and secretion of various cytokines [18] and also the migration of mouse dendritic cells in vitro [19]. Moreover, knockout of the α4 subunit of GABA(A)Rs has been seen to enhance lung inflammation and airway reactivity in a murine asthma model [20]; purportedly mediated by excessive activation of T cells. These multiple findings suggest GABA can function as an immunosuppressive molecule mediated through GABA(A)Rs.

However, despite these reports the topic remains controversial. While several studies have reported changes in the functioning of the immune system in response to either GABA or GABA(A)R allosteric modulators, these responses vary in intensity, indicating a reproducibility issues in working with these molecules. Similarly, reports have disagreed on the specific subunit expression of GABA(A)R on immune cells. In addition, positive allosteric modulators such as benzodiazepines and especially diazepam [21] are also capable of activation of TSPO, a peripheral benzodiazepine receptor located in mitochondria. At least some of the effects of immunosuppressive GABAergic signalling identified by benzodiazepine treatments could therefore instead be attributed to the modulation of TSPO function, as opposed to GABAergic signalling and hence further studies are required to determine which effects on the immune system are elicited by TSPO or GABA(A)R.

This report, therefore, aimed to identify the GABAergic effects on cells of the murine and human immune systems and to discern these from potential influences of other mechanisms.

## Materials and methods

### PBMC and splenocyte isolations

Human B cells, T cells and monocytes were purified from human PBMCs obtained from healthy donor leukocyte cones through Southampton National Blood Services with prior informed consent and Ethical approval from the East of Scotland Research Ethics Service, Tayside, UK. REC Reference: 16/ES/0048. All animal experiments were conducted under UK Home Office licence number P81E129B7 following approval by local ethical committees, reporting to the Home Office Animal Welfare Ethical Review Board (AWERB) at the University of Southampton.

#### PBMC isolation

Anonymised leukocyte cones were obtained with consent from the National Blood Service (Southampton, UK) and were used within 4 hours for preparation of PBMCs by density gradient centrifugation (Lymphoprep; Stemcell Technologies, Cambridge, UK). Residual red blood cells were removed through addition of Ammonium-Chloride-Potassium lysing buffer (ThermoFisher Scientific, Massachusetts, USA), and contaminating platelets eliminated by three slow speed centrifugations (200g, 10 minutes), in Roswell Park Memorial Institute (RPMI) 1640 medium (Sigma-Aldrich, Dorset, UK). PBMC were resuspended in freezing medium (composed of 90% (v/v) foetal calf serum (FCS) and 10% (v/v) dimethyl sulfoxide (DMSO) (Sigma-Aldrich)), initially frozen at -80°C and then subsequently transferred to liquid nitrogen for extended storage.

Several PBMC samples were used for isolation of the subsets of immune cells. Monocytes, T cells, B cells and NK cells were isolated using the following kits from Miltenyi Biotec: Pan Monocyte Isolation Kit (Cat. No 130-096-537), Pan T Cell Isolation Kit (Cat. No 130-096-535), Pan B Cell Isolation Kit (Cat. No 130-101-638) and NK Cell Isolation Kit (Cat. No 130-092-657) respectively. The cells were isolated according to the manufacturer’s protocols.

#### Splenocyte isolation

Splenocytes were isolated from female wildtype BALB/c mice spleens by using a cell strainer, and subsequent collection of the cells in phosphate buffered saline (PBS). Residual red blood cells were removed through addition of Ammonium-Chloride-Potassium lysing buffer, before the remaining splenocytes were resuspended in RPMI 1640 medium containing 10% FCS, L-glutamine, pyruvate, the antibiotics penicillin and streptomycin and 2μM 2-Betamercaptoethanol (Sigma-Aldrich).

Several splenocyte samples were further processed to isolate CD4^+^ and CD8^+^ cells using the following kits from Stemcell Technologies: EasySep™ Mouse CD4 Positive Selection Kit II (Cat. No 18952RF) and EasySep™ Mouse CD8^+^ T Cell Isolation Kit (Cat No 19853RF). The cells were isolated according to the manufacturer’s protocols.

### RT-PCR

Isolated cells were used for total RNA preparation using the Monarch Total RNA Miniprep Kit from New England Biolabs (NEB Cat. No. T2010S) according to the manufacturer’s protocol. Subsequently, 1μg of total RNA was used to prepare cDNA with LunaScript™ RT SuperMix Kit (NEB Cat. No. E3010S). The resulting cDNA was used to detect GABA(A)R subunits with primers as described in [13].

### Proliferation assays

PBMCs and splenocytes were isolated as described above, and resuspended in PBS at a density of 1x10^7^ cells/ml. Cells were then stained with 5μM Carboxyfluorescein succinimidyl ester (CFSE; Biolegend, San Diego, USA), and incubated at room temperature for 10 minutes, protected from light. RPMI medium, containing 10% FCS, L-glutamine, pyruvate and the antibiotics penicillin and streptomycin was added and cells were centrifuged for 5 minutes at 300g before being resuspended in RPMI medium containing 10% FCS, L-glutamine, pyruvate and the antibiotics penicillin and streptomycin. PBMC were plated in 96 well plates, 50 000 cells per well with 150 μl of medium, prior to commencement of the proliferation assay as previously described [22].

Cells were activated with soluble anti-CD3 antibody (anti-human CD3: clone OKT3; anti-mouse CD3: clone 145.2C11; both in house and with <10 EU/mg endotoxin) at concentrations indicated in individual figure legends. In addition, GABA, alprazolam, diazepam, allopregnanolone, BMI and TPMPA (all from Sigma-Aldrich) were used as activating or inhibitory reagents within the assay. The concentrations of each reagent used within individual experiments are indicated in the text.

PBMC were then incubated for 96 hours, while splenocytes were incubated for 48 hours. Cells were then harvested, and the percentage of proliferating cells in each condition determined through flow cytometry, as described below.

### Flow cytometry

The following antibodies were used for flow cytometry: mouse anti-human APC-CD8(SK1), mouse anti-human PE-CD4(OKT4), rat anti-mouse PE-CD4(GK1.5) and rat anti-mouse APC-CD8(53–6.7), in addition to the appropriate isotype controls (all from Biolegend). Cells were harvested and washed in flow cytometry buffer (PBS supplemented with 1% (w/v) BSA, 0.1% (w/v) sodium azide and 0.5mM EDTA (all Sigma-Aldrich)), before being stained with fluorochrome-conjugated antibodies according to the manufacturer’s instructions. Following staining, cells were washed three times in flow cytometry buffer, before being fixed with 1% (w/v) paraformaldehyde (BD Biosciences, Oxford, UK).

Flow cytometry was performed on a FACSCalibur using BD Cellquest software, or on a FACSCanto-II using BD FACSDiva software. Further analysis and figure preparation was carried out using FlowJo version 10 software.

### Confocal imaging

Human PBMC’s or mouse splenocytes were fixed with 4% paraformaldehyde before incubating with GABA receptor subunit antibodies: GABRR2 (ab83223), GABRA1 (ab33299) or GABRP (ab26055)(all Abcam). Subunit antibodies were detected with Alexa Fluor 647 goat anti-rabbit secondary (Thermo Fisher/Invitrogen). PBMC’s were double labelled with Alexa Fluor 488-conjugated mouse anti-human CD3 (Biolegend). Cells were counterstained with DAPI, transferred to chambered coverslips (Ibidi) and confocal images acquired with the Nanoimager S Mark II from ONI (Oxford Nanoimaging) running under NimOS software (Version 1.4.3, ONI) with lasers; 405nm/150mW, 488nm/1W and 640nm/1W and dual emission channels split at 640nm.

### Statistical analysis

Experimental statistical analyses were performed using GraphPad prism software (version 8). One-way or two-way ANOVA or Student’s paired t-test were used throughout, as indicated in the text. Data were considered statistically significant when p = <0.05.

## Results

### GABA(A)Rs are expressed in human and mouse immune cells

GABA(A)R subunits form major inhibitory sites in the CNS. Various reports have also described their expression in immune cells [11–13, 23]. However, there are discrepancies in assessing the expression profiles of GABA(A)Rs and not all studies included all subunits. Therefore, in order to comprehensively establish the expression of the different GABA(A)R subunits in various mouse and human immune cells we performed RT-PCR using previously described and validated primer sets [13]. We also assessed expression of the two enzymes, GAD65 and GAD67 that are responsible for GABA synthesis.

**Fig 1:**
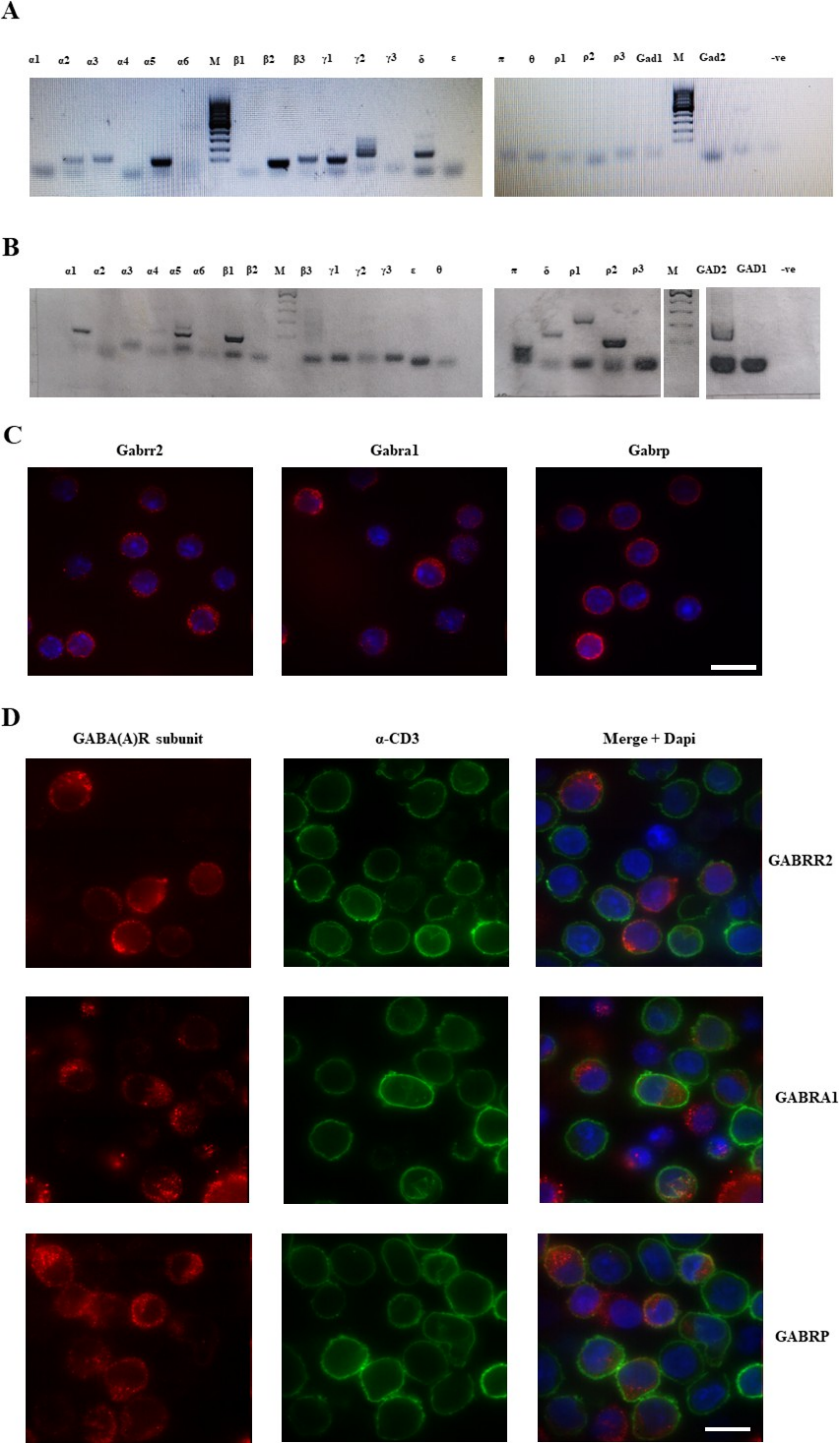
Expression of GABA(A)R subunits in mouse and human T cells. **A**. Representative gel of PCR to amplify GABA(A)R subunits from mouse CD8^+^ cells. DNA ladder is GeneRuler 100 bp DNA Ladder (ThermoFisher, Cat. No. SM0244) **B**. Representative gel of PCR to amplify GABA(A)R subunits from human T cells. DNA ladder is GeneRuler 100 bp DNA Ladder (ThermoFisher, Cat. No. SM0244) **C**. Staining of mouse splenocytes for Gabrr2, Gabra1 and Gabrp subunits. Red–staining of the subunits, blue–DAPI. **D**. Staining of human PBMCs for the GABRR2, GABRA1 and GABRP (red), anti-CD3 (green) and DAPI (blue). Scale bars 10μm.

Total RNA was isolated from the purified fractions of human and mouse immune cells (CD4^+^ and CD8^+^ mouse T cells; human T cells, B cells, monocytes and NK cells) and reverse-transcribed. The resulting cDNA was subsequently amplified using specific primer pair for each receptor subunit and assessed on an agarose gel. Representative agarose gels displaying the expression of various GABA(A)R subunits in mouse CD8^+^ cells and human T cells are shown in Fig 1A and 1B respectively. Experiments were performed at least three times with similar results and are summarized in Table 1.

**Table 1:** Expression levels of GABA(A)R subunits in various types of immune cells.

Subunit/Cell type	Mouse CD4+	Mouse CD8+	Human T cells	Human B-cells	Human monocytes	Human NK cells
α1	-	-	++	++	+	-
α2	++	+	-	-	-	-
α3	++	++	-	-	-	-
α4	-	-	+	+	+	+
α5	+++	+++	++	-	-	-
α6	-	-	-	-	-	-
β1	-	-	++	++	++	-
β2	+++	++	+	++	-	-
β3	+++	+	+	-	-	-
γ1	++	++	-	-	-	-
γ2	+	+	-	-	-	-
γ3	-	-	-	-	-	-
δ	+++	+++	++	-	+	-
ε	-	-	-	-	-	-
π	-	-	++	++	++	++
θ	-	-	-	-	-	-
ρ1	-	-	++	-	+	++
ρ2	-	-	+++	++	++	++
ρ3	-	-	-	-	-	-

The expression levels indicated are based on the thickness and intensity of the bands in the gel. “+++” represents high level of expression, “++”—medium, “+”—weak expression and “- “–expression not detected.

We found the expression levels of GABA(A)R subunits to be markedly different in the various mouse and human immune cells. The major subunits in mouse CD4^+^ and CD8^+^ T cells were α2, α3, α5, β2, β3, and γ1. No GAD65 or GAD67 were detected. Human T cells mainly expressed α1, α5, β1 and π subunits. The notable difference between mouse and human T cells was high expression of ρ2 and to lesser extent of ρ1 subunits within human T cells. Indeed, ρ2 subunit expression is a prominent feature of human T cells making it a primary target for further investigation. Another notable difference between human and mouse T cells was the expression of GAD2 that encodes the GAD65 protein in human T cells. On the whole our data replicated that of previous work [13]. However, the negative results for ρ-subunits as well as for π and α1 subunits in mouse T cells warranted further confirmation and so we investigated protein expression using immunofluorescence. Mouse splenocytes were positive when stained with antibodies against the ρ2 subunit as well as for π and α1 subunits (Fig 1C) which shows a discrepancy between the RT-PCR and immunofluorescence data collected. The immunofluorescence staining for the α1 subunit was cytoplasmic whereas ρ2 and π could be identified at the cell surface as well as in the cytoplasm. There was a clear punctate staining pattern for the ρ2 subunit on the cell surface possibly indicating clustered receptors. We also stained human PBMCs (Fig 1D) to determine whether high gene expression of ρ2 corresponds to high protein expression. Indeed, ρ2 as well as π displayed cell surface and cytoplasmic staining whereas α1 subunit staining was detectable only in the cytoplasm. A notable feature of the above-mentioned subunit staining pattern was its presence on CD3-negative cells, which most likely represent monocytes or B cells. Negative controls for IHC staining are presented in S1 Fig.

### Diazepam inhibits mouse and human T cell proliferation via different pathways

Having established the expression of multiple GABA(A)R subunits in mouse and human T cells, we next wished to assess their functionality. The difficulty in assessing the direct action of GABA on immune cells lies in the fact that GABA is present in the blood at variable concentrations (usually 100-500nM depending on health status, age and sex) [24–26]. Therefore, while some reports show a direct action of GABA or the GABA(A)R agonist muscimol on immune cells [15] others do not [14]. To overcome this obstacle, various groups have used positive allosteric modulators to cause stimulation of GABA(A)Rs. One of the most commonly used classes of drugs to stimulate GABA(A)Rs are benzodiazepines, of which diazepam is the most prevalent. We therefore assessed the effect of diazepam on T cell proliferation driven by anti-CD3 stimulation, measured by CFSE dilution.

**Fig 2:**
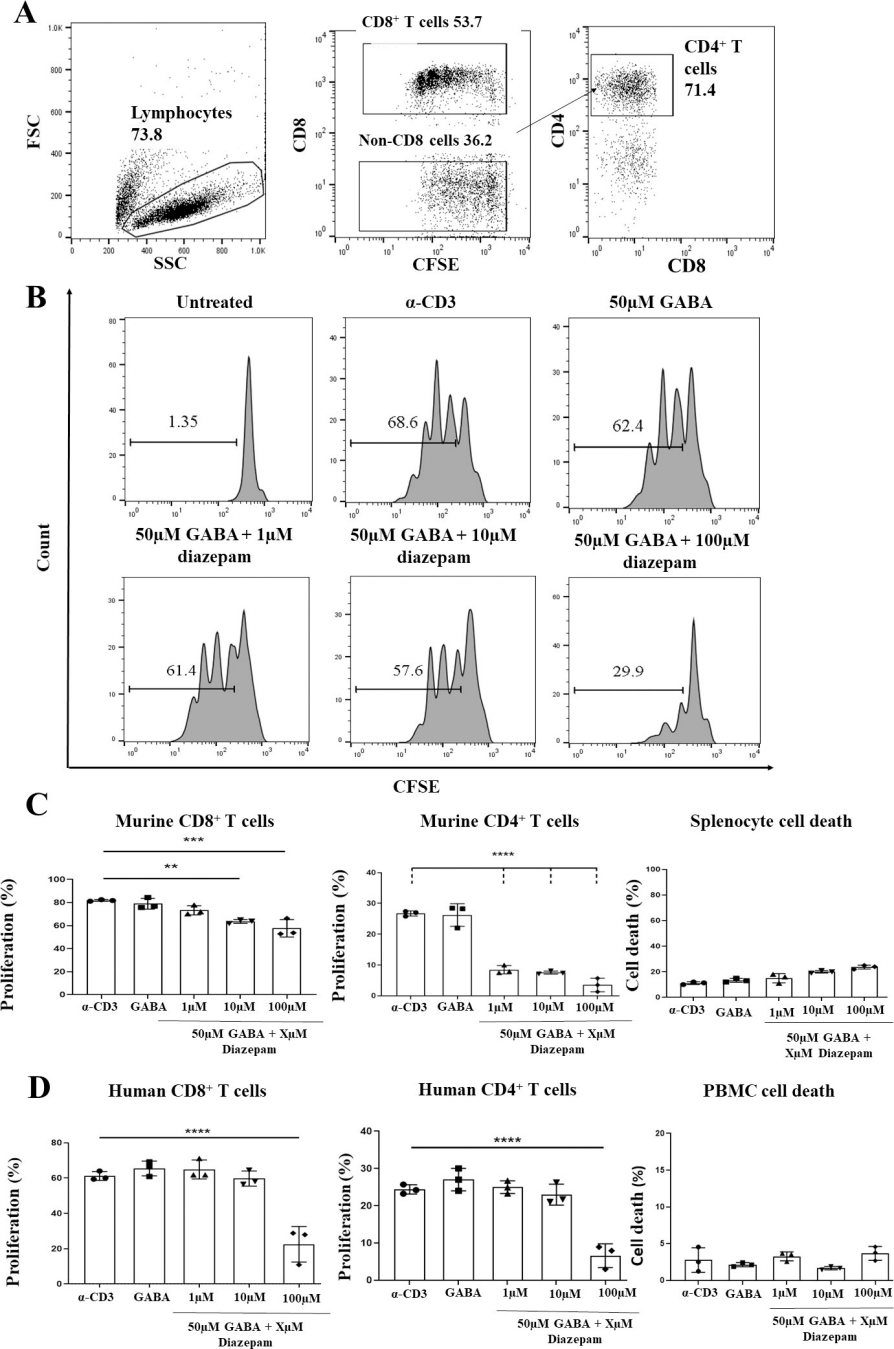
Diazepam can inhibit T cell proliferation in a dose dependent manner. Splenocytes were isolated from the spleens of female wildtype BALB/c mice, and PBMC were isolated from human whole blood samples. Cells were stained with 5 μM CFSE, before being treated with soluble α-CD3 antibody (33 ng/ml for splenocytes, 100 pg/ml for PBMCs), in addition to 50 μM GABA alone, or 50 μM GABA plus 1 μM, 10 μM or 100 μM diazepam. Splenocytes were harvested following 48 hours of treatment, while PBMC were harvested following 96 hours of treatment. The percentage of proliferating T cells in each condition was determined by flow cytometry through assessing the reduction in CFSE fluorescence. **A**. Representative flow cytometry plots depicting the gating strategy used to identify cell populations of interest from PBMC and splenocyte preparations. Lymphocytes were gated according to size (forward scatter; FSC) and granularity (side scatter; SSC). Within the lymphocyte gate, CD8+ and CD4+ T cells were further gated using established lineage markers for these cells (CD8 and CD4). Histograms were used to determine the percentage of proliferating cells present under each condition, compared to an untreated control. **B**. Exemplar flow cytometry plots (from human CD8+ T cells) depicting the dose dependent inhibition of α-CD3 stimulated proliferation caused by treatment of cells with 50 μM GABA and varying concentrations of diazepam. **C**. Dose dependent inhibition of α-CD3 stimulated proliferation in CD8+ and CD4+ T cells following treatment of splenocytes with 50 μM GABA and varying concentrations of diazepam. In addition, splenocyte death in response to diazepam treatment is shown. **D**. Dose dependent inhibition of α-CD3 stimulated proliferation in CD8+ and CD4+ T cell following treatment of PBMC with 50 μM GABA and varying concentrations of diazepam In addition, PBMC death in response to diazepam treatment is shown. Data shown are from 3 independent experiments, with error bars (SD). Differences between groups were assessed by one-way ANOVA. * = p<0.05. ** = p<0.01. *** = p<0.001. **** = p<0.0001.

Examples of flow cytometry gating strategy are shown in Fig 2A and 2B. Our data show that mouse T cell proliferation was effectively inhibited by diazepam treatment at a concentration of 100 μM (Fig 2C) with CD4^+^ cells more susceptible than CD8^+^. The effective concentration of diazepam that evoked inhibition in mouse CD4^+^ T cells was 1 μM. Cell death was modestly affected by increasing concentrations of diazepam rising from around 15% for 1 μM diazepam to more than 20% for 100 μM. In contrast, human T cells did not show inhibition of proliferation at doses less than 100μM and there were no marked differences between CD4^+^ and CD8^+^ responses (Fig 2D). The cell death was low (3%) and unaffected by rising diazepam concentrations.

**Fig 3:**
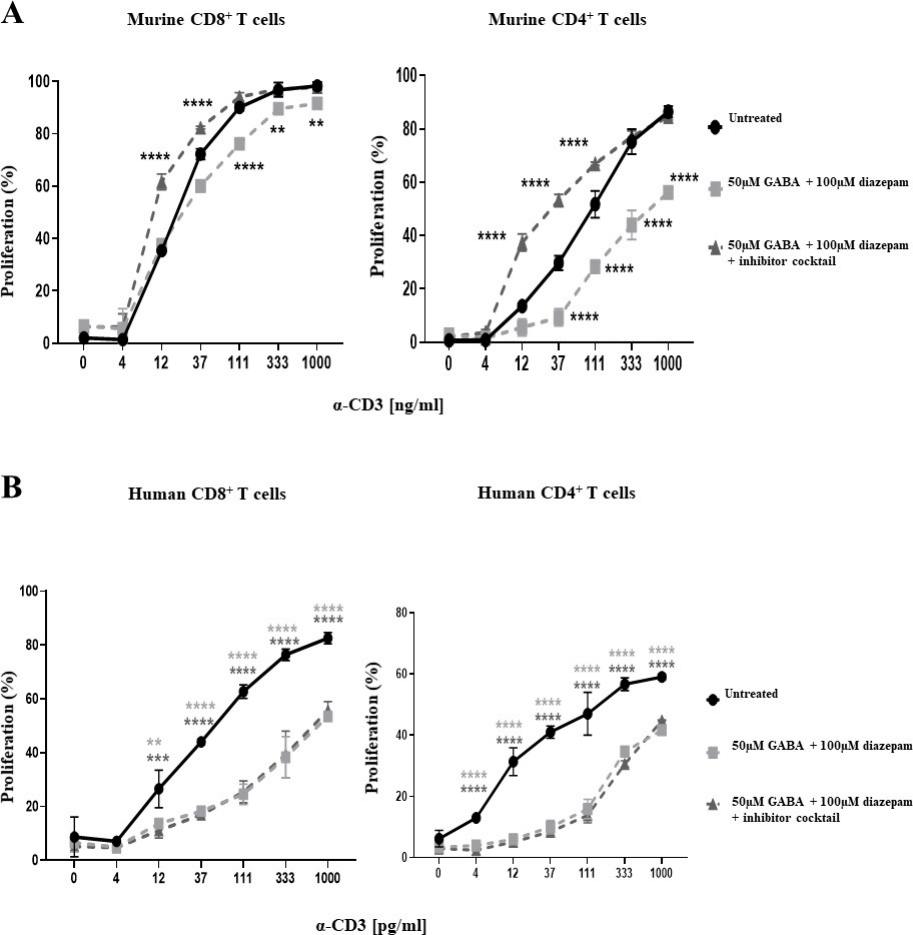
Diazepam inhibits mouse and human T cell proliferation via different pathways. Splenocytes and PBMC were treated with α-CD3, 50 μM GABA, 100 μM diazepam, and a cocktail of GABA(A)R inhibitors consisting of 100 μM BMI and 50 μM TPMPA. The reduction in proliferation under each condition as compared to a positive control of α-CD3 alone was assessed by flow cytometry following 48 hours of treatment for splenocytes, and 96 hours of treatment in PBMC. **A**. Treatment with the GABA(A)R inhibitor cocktail is able to rescue CD8+ and CD4+ T cell proliferation in murine T cells. **B**. Treatment cannot rescue proliferation in humans T cells. Data shown is from one representative experiment, with error bars (SD). Differences between groups were assessed by one-way ANOVA. * = p<0.05. ** = p<0.01. *** = p<0.001. **** = p<0.0001.

As discussed earlier, the effects of diazepam could be attributed either to GABAergic signalling or TSPO activation. To distinguish these mechanisms, we performed rescue experiments adding a cocktail of inhibitors of GABA(A)Rs (100 μM BMI + 50 μM TPMPA) along with 50 μM of GABA and 100 μM diazepam. (Fig 3A and 3B). The inhibition of T cell proliferation by diazepam observed in mouse T cells could be rescued by adding the cocktail of GABA(A)R antagonists (Fig 3A). The same cocktail also overcame the excessive cell death of murine T cells given 100 μM diazepam. This same rescue was not replicated in human T cells (Fig 3B). These data strongly suggest that the immunosuppressive actions of diazepam are mainly mediated through GABA(A)R activation in mouse T cells but that in human T cells its effects are mediated by other mechanisms, possibly through TSPO binding.

### Role of GABA(A)Rs in human and mouse T cell proliferation

**Fig 4:**
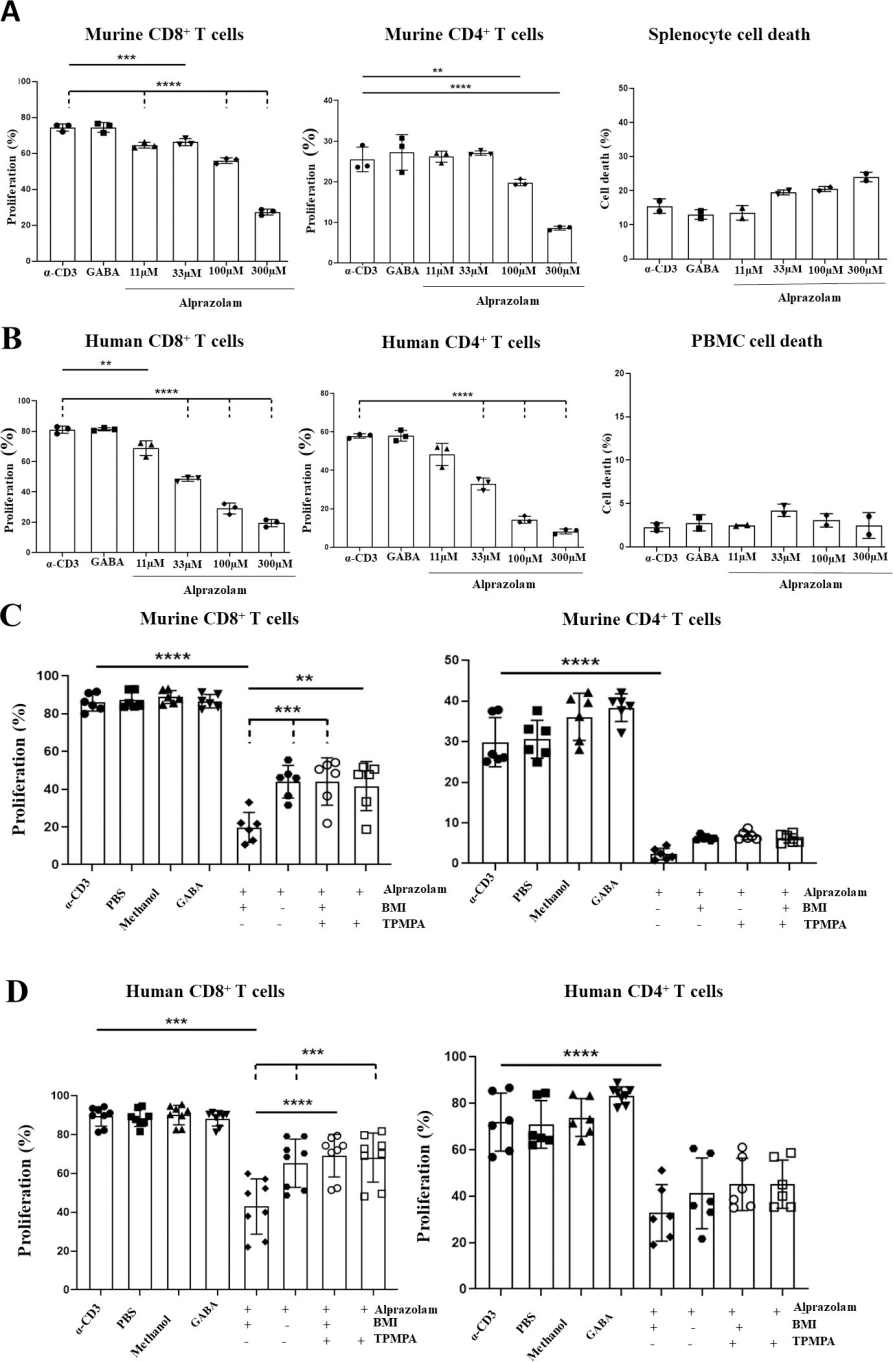
Alprazolam causes suppression of human and mouse T cell proliferation, which can be recovered by multiple GABA(A)R inhibitors. **A, B)** Splenocytes were isolated from the spleens of female wildtype BALB/c mice, and PBMCs were isolated from human whole blood samples. Cells were stained with 5 μM CFSE, before being treated with soluble α-CD3 antibody (33 ng/ml for splenocytes, 100 pg/ml for PBMCs), in addition to either 11 μM, 33 μM, 100 μM or 300 μM alprazolam. Splenocytes were harvested following 48 hours of treatment, while PBMCs were harvested following 96 hours of treatment. The percentage of proliferating T cells in each condition was determined by flow cytometry through assessing the reduction in CFSE fluorescence as compared to a positive control of α-CD3 treatment alone. **A**. Dose dependent inhibition of CD8^+^ and CD4^+^ T cell proliferation by alprazolam in treated splenocytes, in addition to total splenocyte death in response to alprazolam treatment. **B**. Dose dependent inhibition of CD8^+^ and CD4^+^ T cell proliferation by alprazolam in treated PBMCs, in addition to total PBMC death in response to alprazolam treatment. **C, D)** Splenocytes and PBMCs were treated with α-CD3 (33 ng/ml for splenocytes, 100 pg/ml for PBMCs), 100 μM (for mouse) or 33 μM (for humans) alprazolam, and either a cocktail of GABA(A)R inhibitors consisting of 100 μM BMI and 50 μM TPMPA, or each individual inhibitor alone. The reduction in proliferation under each condition as compared to a positive control of α-CD3 alone was assessed by flow cytometry following 48 hours of treatment in splenocytes, and 96 hours of treatment in PBMCs. **C**. Treatment with the GABA(A)R inhibitor cocktail is able to rescue CD8^+^ and CD4^+^ T cell proliferation in murine T cells. **D**. Treatment with the GABA(A)R inhibitor cocktail is able to rescue CD8^+^ and CD4^+^ T cell proliferation in human T cells. Data shown are from 3 independent experiments, with error bars (SD). Differences between groups were assessed by one-way ANOVA. * = p<0.05. ** = p<0.01. *** = p<0.001. **** = p<0.0001. UN = untreated.

To further decipher the role of GABAergic signalling in T cell proliferation and to exclude possible effects of TSPO modulation we next investigated the effects of the benzodiazepine alprazolam. Alprazolam positively modulates GABA(A)Rs but has been shown to have no effect on TSPO [27]. Performing similar proliferation assays as before in the presence and absence of alprazolam showed that alprazolam can inhibit T cell proliferation in both human PBMCs and mouse splenocytes in a dose-dependent manner (Fig 4A and 4B). Interestingly, the effective concentrations of alprazolam required were higher in mouse T cells than humans (100 μM vs 33 μM respectively). This is possibly due to the different subunit composition described in Table 1. Alprazolam also displayed higher toxicity for mouse T cells than for human (Fig 4A and 4B).

To counter the effects of alprazolam-mediated GABAergic signalling on T cell proliferation we again used a cocktail of GABA(A)R antagonists containing BMI and TPMPA. The rationale for the use of TPMPA, the antagonist specific for ρ-containing receptors, is that these receptors have been shown to be present on the cell membranes of mouse and human T cells. As GABA has been shown to be present in culture medium being secreted by immune cells [14] it may be necessary to inhibit all GABAergic signalling to counter reduced T cell proliferation due to activation of GABA(A)Rs. As shown in Fig 4C and 4D, suppression of T cell proliferation in response to treatment with alprazolam could be rescued by treatment with a combination of BMI and TPMPA both in mouse and human T cells. The effect was much more pronounced and reached statistical significance in CD8^+^ cells while in CD4^+^ cells there was a more subtle trend towards recovery. In addition to reversal of suppression in response to treatment with BMI and TPMPA together, we also showed that suppression of T cell proliferation in response to alprazolam could be rescued by BMI treatment alone with both mice and human cells (Fig 4C and 4D). Surprisingly however, the rescue effect of TPMPA alone was similar to that seen with BMI alone and the combination of BMI and TPMPA.

### Allopregnanolone inhibits human and mouse T cell proliferation

To further explore the role of the GABA(A)R in T cell activation leading to proliferation we studied the effects of the neurosteroid allopregnanolone on human and murine T cells. Neurosteroids are implicated in various processes in the CNS [28–30]. It has been shown that allopregnanolone potentiates the actions of αβ-containing GABA(A)Rs at nanomolar concentrations and ρ-containing receptors at micromolar concentrations [31, 32].

**Fig 5:**
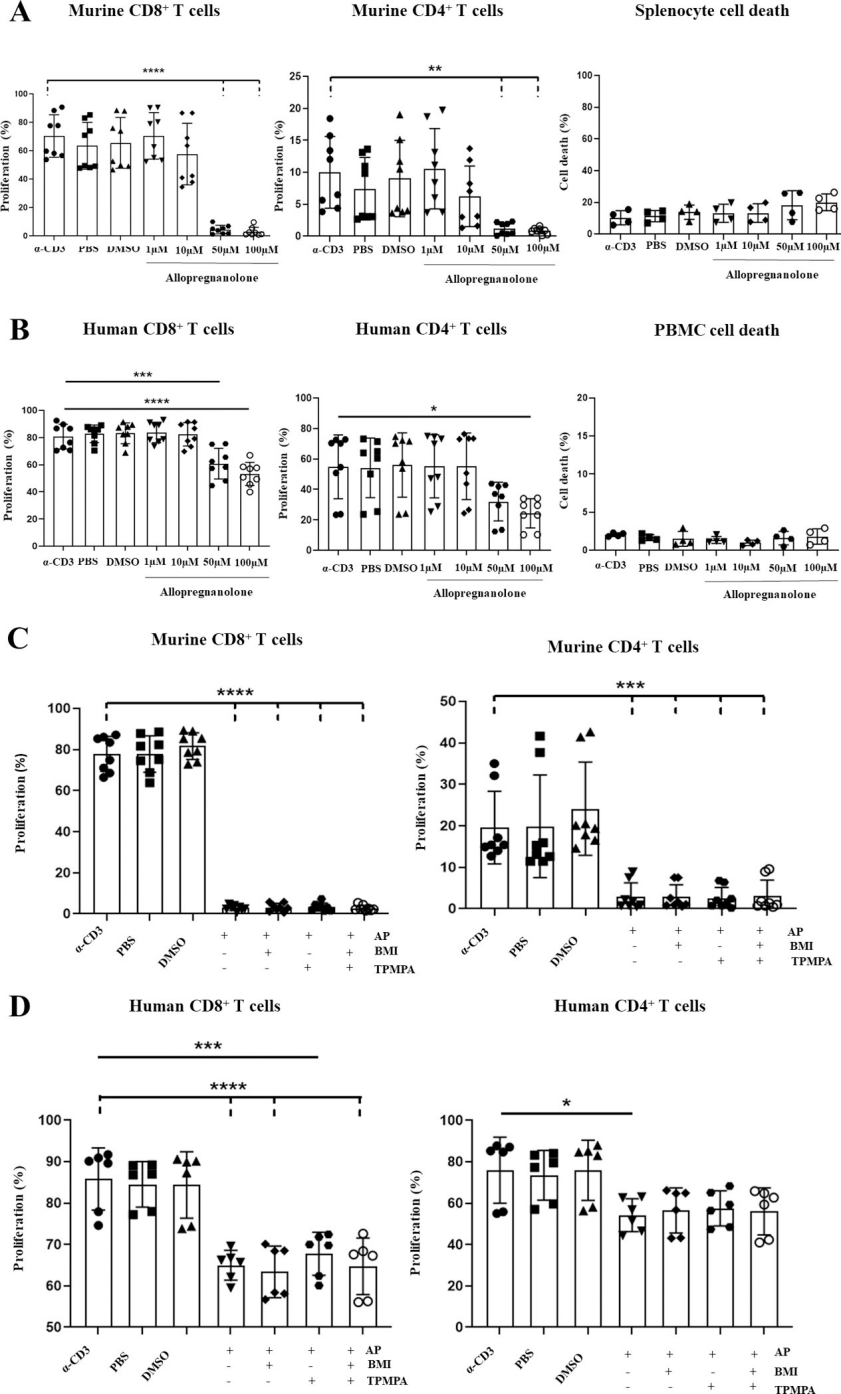
Allopregnanolone inhibits human and mouse T-cell proliferation, which cannot be rescued with GABA(A)R inhibitors. **A, B)** Allopregnanolone causes a dose dependent inhibition of T cell proliferation in mouse and humans. Splenocytes were isolated from the spleens of female wildtype BALB/c mice, and PBMCs were isolated from human whole blood samples. Cells were stained with 5 μM CFSE, before being treated with soluble α-CD3 antibody (33 ng/ml for splenocytes, 100 pg/ml for PBMCs), in addition to either 1 μM, 10 μM, 50 μM or 100 μM allopregnanolone. Splenocytes were harvested following 48 hours of treatment, while PBMCs were harvested following 96 hours of treatment. The percentage of proliferating T cells in each condition was determined by flow cytometry through assessing the reduction in CFSE fluorescence as compared to a positive control of α-CD3 treatment alone. **A**. Dose dependent inhibition of CD8^+^ and CD4^+^ T cell proliferation by allopregnanolone in treated splenocytes, in addition to total splenocyte death in response to allopregnanolone treatment. **B**. Dose dependent inhibition of CD8+ and CD4+ T cell proliferation by allopregnanolone in treated PBMCs, in addition to total PBMC death in response to allopregnanolone treatment. **C, D**) Treatment with a combination of BMI and TPMPA cannot rescue allopregnanolone induced inhibition of proliferation in mice or humans. Splenocytes and PBMCs were treated with α-CD3 (33 ng/ml for splenocytes, 100 pg/ml for PBMCs), 50 μM allopregnanolone, and either a cocktail of GABA(A)R inhibitors consisting of 100 μM BMI and 50 μM TPMPA, or each individual inhibitor alone. The reduction in proliferation under each condition as compared to a positive control of α-CD3 alone was assessed by flow cytometry following 48 hours of treatment in splenocytes, and 96 hours of treatment in PBMCs. **C**. No recovery of allopregnanolone-induced inhibition of proliferation in response to treatment with BMI, TPMPA, or a combination of both inhibitors in splenocytes. **D**. No recovery of allopregnanolone-induced inhibition of proliferation in response to treatment with BMI, TPMPA, or a combination of both inhibitors in PBMCs. Data shown are from at least 3 independent experiments, with error bars (SD). Differences between groups were assessed by one way ANOVA. * = p<0.05. ** = p<0.01. *** = p<0.001. **** = p<0.0001. UN = untreated. AP = allopregnanolone.

We found that allopregnanolone could effectively inhibit both mouse and human T cell proliferation at a concentration of 50 μM (Fig 5A and 5B). Interestingly, 1 μM and 10 μM concentrations of allopregnanolone failed to have any effect on either mouse or human T cell proliferation except with mouse CD4^+^ cells. Given that the sensitivity of αβ-containing receptors is in the nanomolar range we speculate that the inhibition of T cell proliferation by allopregnanolone is mediated predominantly by ρ-containing receptors. Our data also show that the viability of mouse splenocytes was more affected by allopregnanolone treatment than human PBMCs. The mechanism of increased cell death in mouse splenocytes after allopregnanolone treatment warrants further investigation. Interestingly, the allopregnanolone-mediated inhibition of T cell proliferation could not be rescued by treatment with the cocktail of BMI and TPMPA (Fig 5C and 5D). The lack of rescue of T cell proliferation inhibition may be due to differences in the binding sites of allopregnanolone and GABA(A)R antagonists.

## Discussion

The immunosuppressive properties of GABA and its mediation through GABA(A)Rs has been explored for some time, reviewed recently by Prud’homme et al., 2015 [33]. The results are diverse and largely based on pharmacological interventions which can be subject to alternative interpretation. For example, some research emphasizes the immunosuppressive role of GABA(A)R activation while others attribute this immunosuppression to non-specific, off-target effects of the drugs. Indeed, several of the common pharmacological agents used to activate GABA(A)Rs also affect other signalling molecules. One of the most common GABA(A)R modulators, diazepam, inhibits various immune functions and increases susceptibility to infection [34–37]. Diazepam is a good example of the difficulties arising from interpretation of pharmacological inhibitors as it binds to both the GABA(A)Rs and the peripheral benzodiazepine receptor, TSPO. Indeed, TSPO has been shown to bind diazepam with high affinity and hence the immunomodulatory action of diazepam may be explained by this binding and subsequent effects on steroid metabolism [38].

Our experiments uncovered a difference in the mechanisms of action of diazepam on mouse and human immune cells. These differences may explain the discrepancies in diazepam data interpretation reported by previous researchers [39, 40].

According to our data, murine CD4+ cell proliferation is inhibited by as little as 1 μM of diazepam while CD8+ cells showed clear signs of inhibition starting at 10 μM (Fig 2C). This difference between CD4+ and CD8+ cells may be attributed to variation in their subunit expression. Indeed, according to our data (Table 1) the expression of β subunits in CD8+ cells was lower when compared to CD4+ T cells. We speculate that this may give rise to a subpopulation of binary receptors containing only α and γ subunits. According to the observation of Granja et al., 1997 [41] αγ- receptors may exist, bind to GABA and to diazepam. However, binding of diazepam to the binary αγ-receptors was approximately 3 times weaker than to the ternary αβγ-receptors. The inhibition of proliferation could be rescued by antagonist treatment (Fig 3A). Diazepam therapeutic blood concentrations in humans are reported to be in the range of 0.1–1 mg/L [42], corresponding to 0.35–3.5 μM. As mouse T-cells respond to therapeutic concentrations of diazepam and the inhibition of proliferation could be rescued by antagonist treatment, we conclude that effects of diazepam in mouse T cells are mediated mainly by GABA(A)Rs.

Human T cells, however, showed less response to diazepam treatment. The inhibition of proliferation trend was noticeable at 10 μM, ~3 times more than therapeutic dose, with statistical significance reached at 100 μM. Certainly, therapeutic concentrations of diazepam may be exceeded in blood of the persons abusing it. To our knowledge, the maximum concentration of diazepam was observed in humans to be as high a 7.6 mg/L corresponding to 26.7 μM [43]. Therefore, although at standard therapeutic dosages diazepam would not affect immune cells in humans, at higher doses found in plasma of substance abusers immunological effects might take place. The inhibition of proliferation we observed in human T cells was not rescued by GABA(A)Rs antagonist treatment.

Our data support the assertion that the effects of diazepam on human T cells are mediated by TSPO activation while effects on mouse T cell proliferation are mediated mainly through GABAergic signalling. This phenomenon may be the source of controversy caused when various research groups have previously assessed the influence of GABA(A)R on immune cells using diazepam and subsequently interpret the evidence either by emphasizing the role of GABA(A)R or by attributing the effects to TSPO. The importance of assessing the immunosuppressive actions of diazepam is emphasized by its popularity as a prescription drug. Indeed, according to the openprescribing.net website there were 4,780,991 diazepam prescriptions in England during the last financial year (2019–2020). Given this huge total there is a clear need to assess the immunological consequences of diazepam for the population. In addition, it is important to note that the immunological effects of diazepam prescriptions could not be mitigated by GABA(A)R blockade outside of the CNS.

To distinguish the effects of GABAergic signalling from those of TSPO in human and mouse cells we used alprazolam, a benzodiazepine that does not bind TSPO. In human cells noticeable inhibition of T cell proliferation was observed at 11 μM becoming statistically significant at 33 μM. T cell proliferation inhibition by alprazolam could be rescued by inhibiting GABAergic signalling. Thus, we can confirm that GABAergic signalling is inhibitory in human T cells as well.

Therapeutic concentrations of alprazolam in human blood plasma are in the range of 10–100 μg/L corresponding to 0.033–0.3 μM [44]. According to Jones and Holmgren [45], the blood plasma concentration of alprazolam in arrested drivers (n = 773) ranged from 0.02–3.9 mg/L corresponding to 0.06–12.6μM. The authors also noted that the alprazolam range in impaired drivers was not different from the range obtained by forensic investigation in deaths attributed to drug intoxication (n = 438) of 0.02–1.6 mg/L. The authors attribute this lack of the difference to the toxicity of co-injested drugs or adverse drug-drug interactions rather than to the effects of alprazolam. Moreover, the authors observed that “in five years of forensic autopsies (~25,000 cases) not a single intoxication death was reported with alprazolam as the only drug present in blood”. This observation makes the establishment of fatal levels of alprazolam difficult. Therefore, it is probably advisable to base our assumptions about the lethal dose of alprazolam on the data provided by FDA where LD50 dose for rats is reported as 331–2171 mg/kg roughly corresponding to 1–7 mM [46]. Our data strongly suggest that at therapeutic levels alprazolam is unlikely to exhibit inhibition of T cell proliferation. However, at levels found in drug abusers some effects are possible.

As expected, alprazolam also inhibited mouse T cell proliferation although at concentrations three times higher than human T cells. The inhibition of proliferation in mouse T cells could also be rescued by GABAergic antagonists.

One the most striking phenomenon observed in our experiments was the fact that benzodiazepine effects were observed without adding GABA to the culture media. Indeed, we have not seen differences in results when the cells are treated with diazepam or alprazolam in the presence or absence of GABA. Based upon literature evidence, we speculate that PBMCs secrete GABA when cultured. According to Bhandage et al., 2020 [47] human and mouse myeloid mononuclear cells are capable of producing GABA. Moreover, Dionisio et al., 2013 [48] identified GAD67, one of the GABA producing enzymes in Jurkat T cells. Our own observations presented on Fig 1B indicate strong expression of GAD2 that encodes the GAD65 enzyme in human T cells. The cells in our experiments were incubated at high density as described in the Materials and Methods. Taking into account our observation of GAD2 expression alongside the observations of others [47, 48] we speculate that the concentration of GABA in the culture medium may reach sufficient levels to act together with benzodiazepines on GABA (A)Rs. It is our intention to investigate this phenomenon further.

The lack of a cumulative effect in rescue when BMI and TPMPA were combined, as compared to the effects of each inhibitor alone was unexpected. BMI acts on αβ-containing GABA(A)Rs whereas TPMPA acts on ρ-containing receptors. Various reports indicate that bicucculine does not act on ρ-containing receptors [49, 50]. If αβ-containing GABA(A)Rs and ρ-containing receptors are confined to separate entities one would expect to observe a cumulative effect of the antagonist cocktail when compared to single antagonist treatments. The lack of cumulative effect of the antagonist cocktail, therefore, implies that both BMI and TPMPA are acting on the same receptor entity. This observation strongly supports the assertion that there are hybrid receptors present on both human and mouse T cells, consisting of both αβ- and ρ (notably ρ2) subunits. The idea that αβ- and ρ subunits may form hybrid receptors exhibiting an intermediate pharmacological profile is in line with the evidence produced by previous studies. Several groups [51–53] have reported that αβ- and ρ subunits indeed may co-assemble and this has been supported by immunoprecipitation, electrophysiological and pharmacological experiments.

Our results add a unique line of evidence supporting the existence of hybrid GABA(A)Rs. Another line of evidence that indicates a prominent role of ρ-containing receptors in these immune cells is the concentration range of benzodiazepines that is required to exert inhibitory effect on T cell proliferation. Diazepam inhibited murine CD8+ T cell proliferation starting at 10 μM and CD4+ proliferation at 1 μM. Human T cells responded only to 100 μM of diazepam. According to [54] ρ-containing receptors are activated by benzodiazepines in the micromolar range (as high as 100 μM for diazepam), whereas alprazolam acted as a negative modulator at the same concentration. This study [54], however, was done on homomeric receptors. It is possible that hybrid receptors containing ρ-subunit may exhibit different pharmacology. Clearly, more research incorporating immunoprecipitation experiments followed by mass spectrometry is needed.

We also studied the effects of allopregnanolone on T cell proliferation. Allopregnanolone is a neurosteroid that is well documented to activate GABA(A)Rs. Allopregnanolone demonstrated reproducible inhibition of mouse and human T cell proliferation at concentrations of about 50 μM and higher. Lower concentrations of 1 μM and 10 μM were ineffective except in mouse CD4^+^ cells. Since nanomolar concentrations of this neurosteroid inhibit mainly αβ-containing receptors we conclude that ρ-containing receptors likely play a significant role in modulating T cell functions.

## Conclusions

In summary, we present several lines of evidence that activation of GABA(A)R in human and mouse T cells inhibits their proliferation. This points to GABAergic signalling mediating suppression of T cell function both in mice and humans. This may open an avenue to design pharmacological interventions based upon GABA(A)R modulation that can evoke either stimulation or suppression of T cell responses.

## Supporting information

S1 Fig Secondary only controls for Fig 1: Expression of GABA(A)R subunits in mouse and human T cells. **A**. Staining of mouse splenocytes with AlexaFluor647-conjugated goat anti-rabbit antibody (Red). Cells were counterstained with DAPI (blue). **B**. Staining of human PBMCs with AlexaFluor488-conjugated mouse anti-CD3 (green) and AlexaFluor647 conjugated goat anti-rabbit antibody (Red). Cells were counterstained with DAPI (blue). Scale bars 10μm.
(TIF)

S1 Raw images (PDF)
