# Supplementary figures and images for: Activation of GABA(A) receptors inhibits T cell proliferation
Source: PLoS One. 2021 May 20;16(5):e0251632. doi: 10.1371/journal.pone.0251632 (PMC8136847; doi:10.1371/journal.pone.0251632)

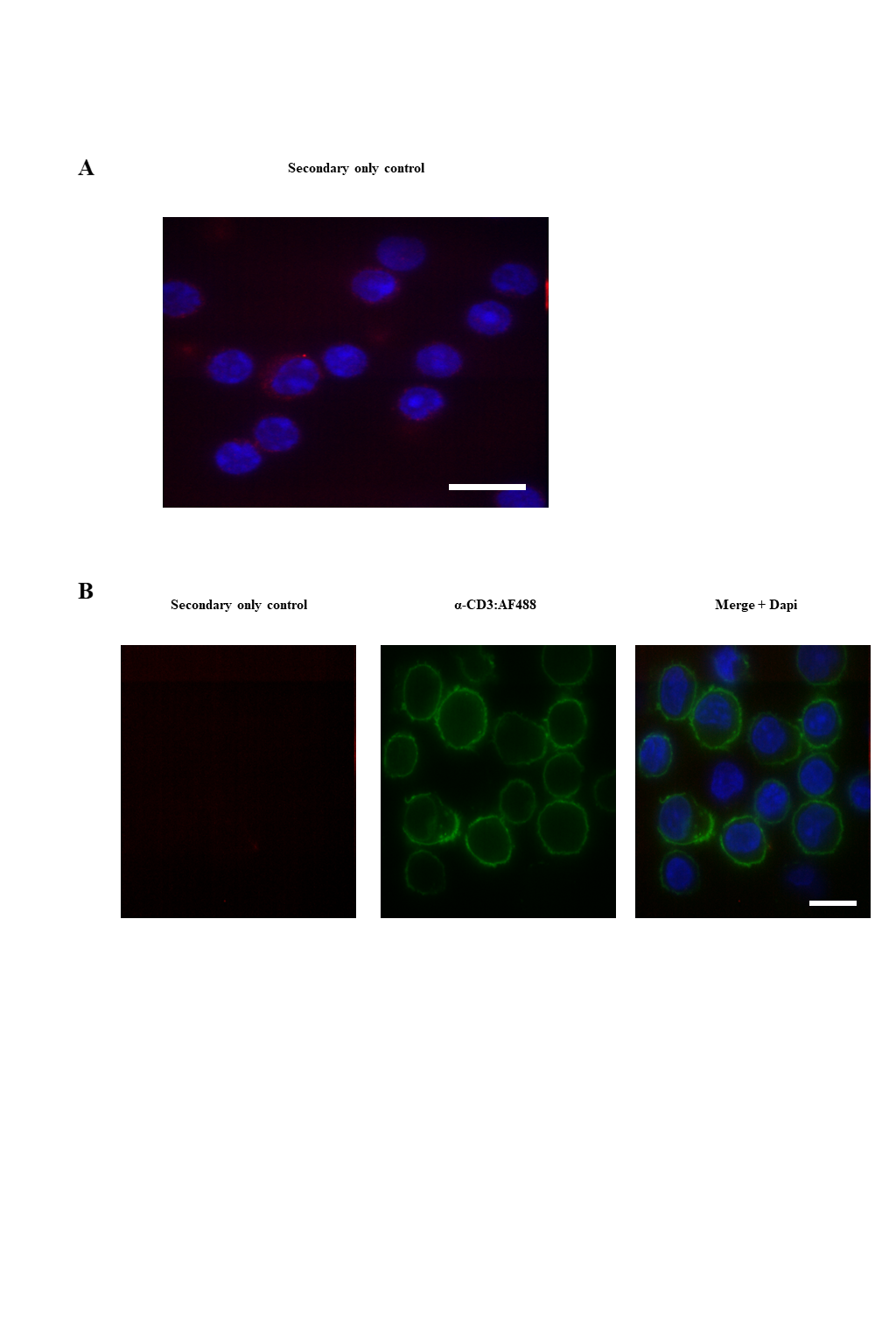

Supplement: S1 Fig — A. Staining of mouse splenocytes with AlexaFluor647-conjugated goat anti-rabbit antibody (Red). Cells were counterstained with DAPI (blue). B. Staining of human PBMCs with AlexaFluor488-conjugated mouse anti-CD3 (green) and AlexaFluor647 conjugated goat anti-rabbit antibody (Red). Cells were counterstained with DAPI (blue). Scale bars 10μm. (TIF) [file pone.0251632.s001.tif]
