# Supplementary material for: Activation of GABA(A) receptors inhibits T cell proliferation
Source: PLoS One. 2021 May 20;16(5):e0251632. doi: 10.1371/journal.pone.0251632 (PMC8136847; doi:10.1371/journal.pone.0251632)

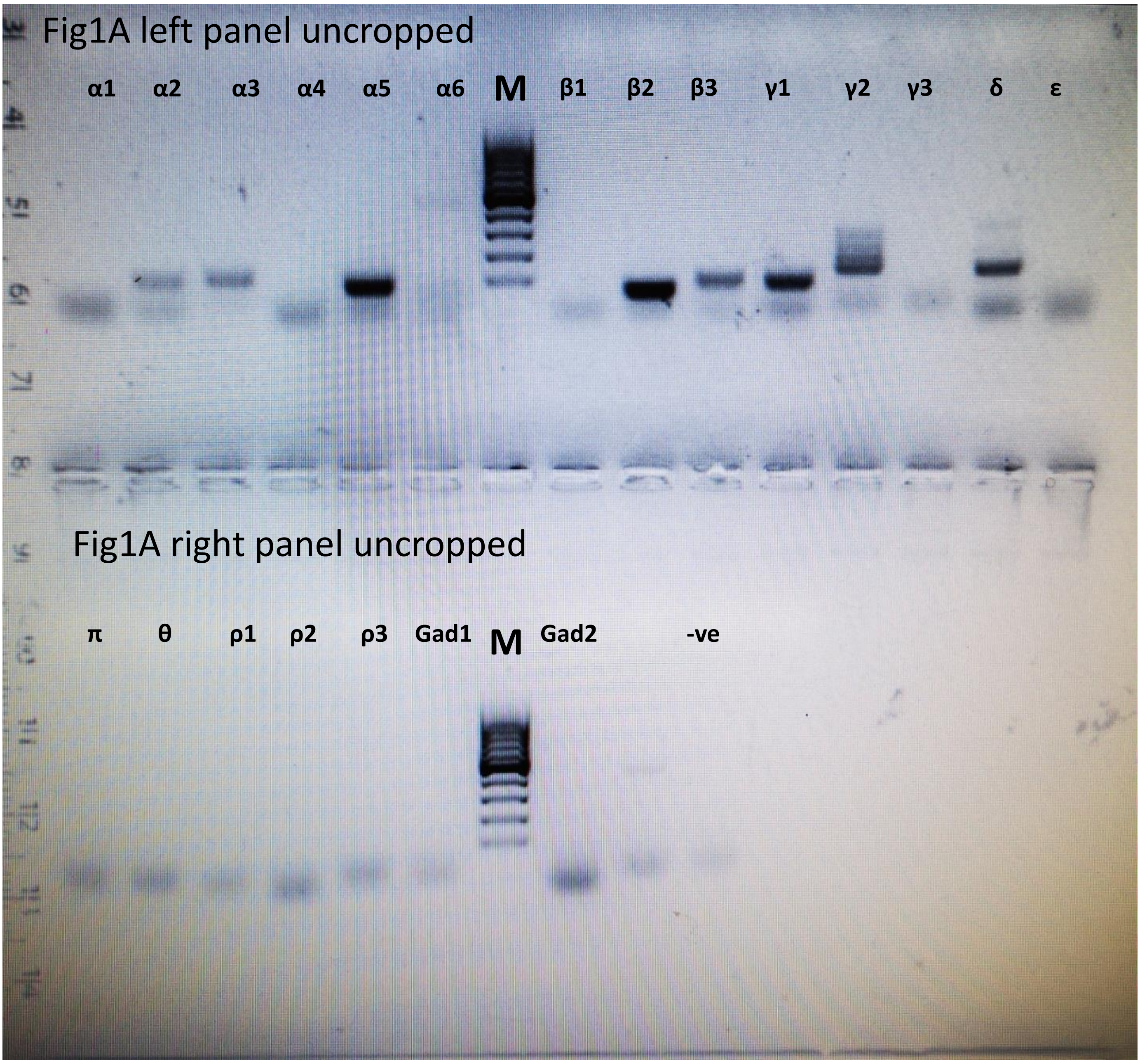
Fig1A left panel uncropped
α1 α2 α3 α4 α5 α6 M β1 β2 β3 γ1 γ2 γ3 δ ε
Fig1A right panel uncropped
π θ ρ1 ρ2 ρ3 Gad1 M Gad2 -ve

Fig1B left panel
uncropped

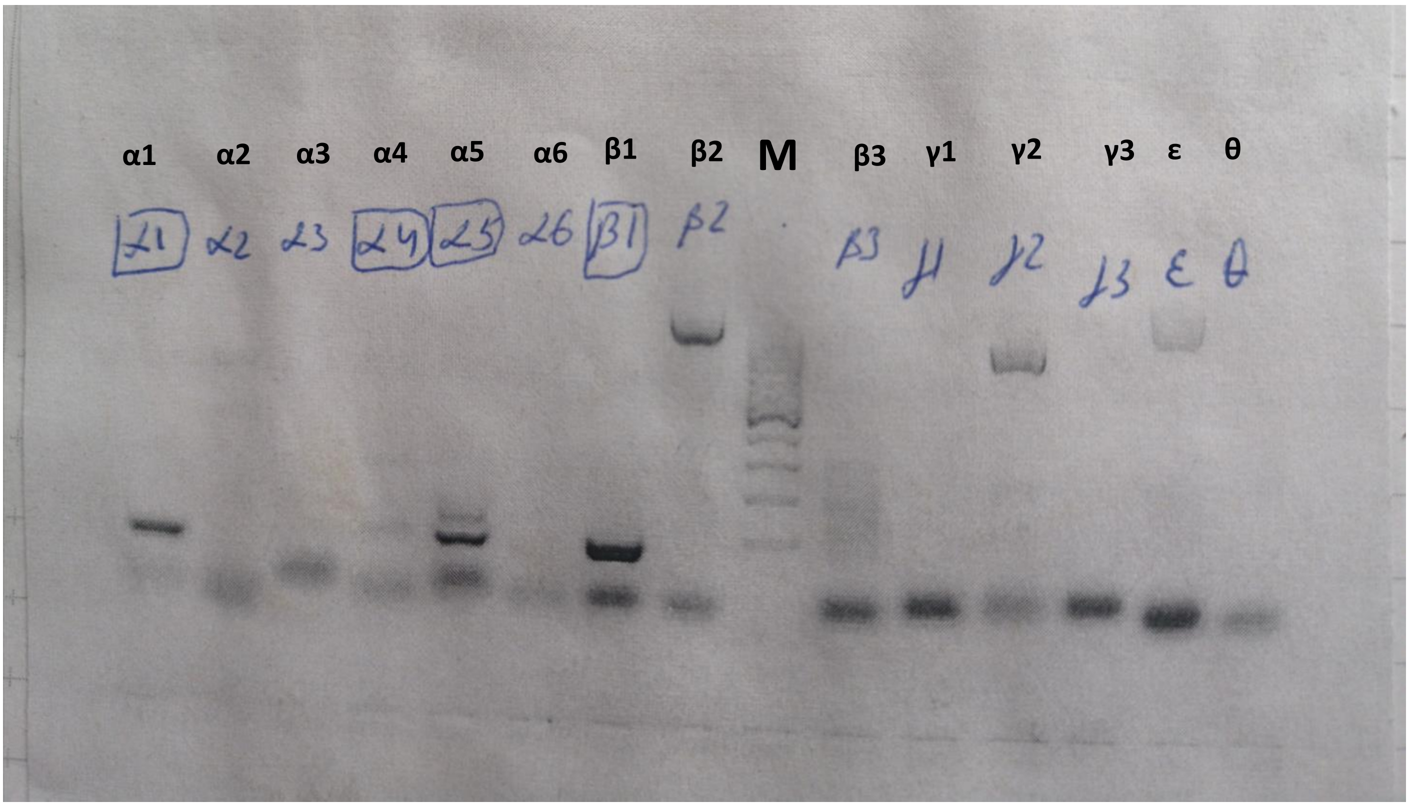

Fig1B right panel
uncropped

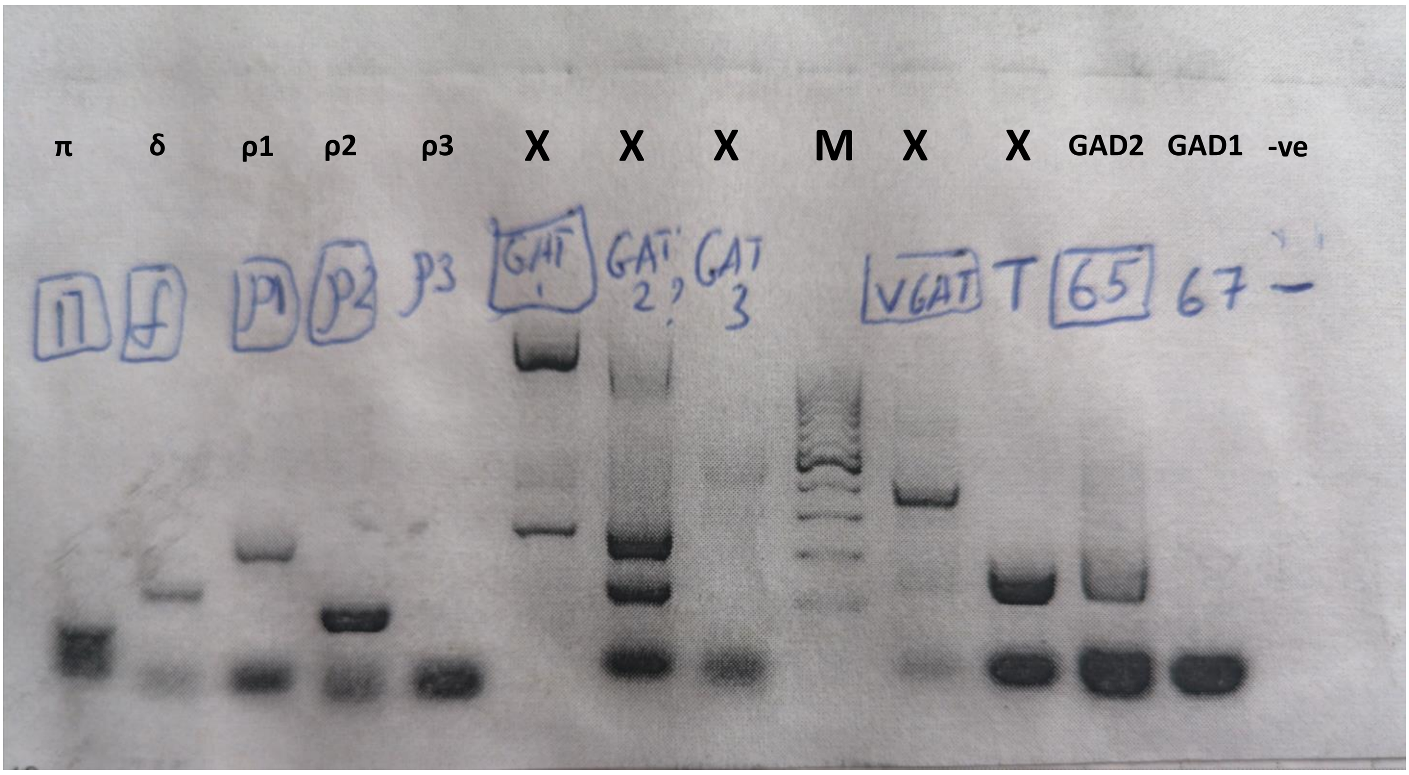

Supplement: S1 Raw images — (PDF) [file pone.0251632.s002.pdf]
